# BARRIERS TO TRANSIT MOBILITY AND WELL-BEING AMONG LATE-LIFE ASIAN INDIAN IMMIGRANTS

**DOI:** 10.1093/geroni/igz038.1332

**Published:** 2019-11-08

**Authors:** Rupal Parekh

**Affiliations:** University of Connecticut, Hartford, Connecticut, United States

## Abstract

Using qualitative interpretative phenomenological analysis and semi-structured interviews, we explored transit mobility needs and its impact on well-being and quality of life among late-life Asian Indian immigrants. Using inductive and deductive methods, we analyzed qualitative data collected from 18 participants. Four themes emerged specific to the influence of contextual factors on transportation mobility barriers among participants. Findings suggest that cultural and individual attitudes combined with the ‘built environment’ hinder participation at the temple and other cultural and religious activities and increase dependence on adult children. The reciprocal fit between transit mobility needs, access to culturally familiar environments and the built environment is critical to freedom, independence, and healthy aging for diverse older adults. Research at the intersection of global aging and transportation mobility requires equal grounding in a range of justice principles (environmental, social, and economic) in the pursuit of sustainable transportation options for diverse older adult populations.

